# Systemic L-Buthionine -S-R-Sulfoximine Treatment Increases Plasma NGF and Upregulates L-cys/L-cys2 Transporter and γ-Glutamylcysteine Ligase mRNAs Through the NGF/TrkA/Akt/Nrf2 Pathway in the Striatum

**DOI:** 10.3389/fncel.2019.00325

**Published:** 2019-07-23

**Authors:** Cesar Valdovinos-Flores, Jorge H. Limón-Pacheco, Renato León-Rodríguez, Pavel Petrosyan, Carla Garza-Lombó, Maria E. Gonsebatt

**Affiliations:** Departamento de Medicina Genómica y Toxicología Ambiental, Instituto de Investigaciones Biomédicas, Universidad Nacional Autónoma de México, Ciudad de México, Mexico

**Keywords:** GSH synthesis inhibition, BSO, brain protection, xCT, Nrf2

## Abstract

Glutathione (GSH) is the most abundant intracellular antioxidant. GSH depletion leads to oxidative stress and neuronal damage in the central nervous system (CNS). In mice, the acute systemic inhibition of GSH synthesis by L-buthionine-S-R-sulfoximine (BSO) triggers a protective response and a subsequent increase in the CNS GSH content. This response might be modulated by a peripheral increment of circulating nerve growth factor (NGF). NGF is an important activator of antioxidant pathways mediated by tropomyosin-related kinase receptor A (TrkA). Here, we report that peripheral administration of BSO increased plasma NGF levels. Additionally, BSO increased NGF levels and activated the NGF/TrkA/Akt pathway in striatal neurons. Moreover, the response in the striatum included an increased transcription of *nrf2*, *gclm*, *lat1*, *eaac1*, and *xct*, all of which are involved in antioxidant responses, and L-cys/L-cys_2_ and glutamate transporters. Using antibody against NGF confirmed that peripheral NGF activated the NGF/TrkA/Akt/Nrf2 pathway in the striatum and subsequently increased the transcription of *gclm*, *nrf2*, *lat1*, *eaac1*, and *xct*. These results provide evidence that the reduction of peripheral GSH pools increases peripheral NGF circulation that orchestrates a neuroprotective response in the CNS, at least in the striatum, through the NGF/TrkA/Akt/Nrf2 pathway.

## Introduction

Reactive oxygen species (ROS) are products of aerobic metabolism. Even in resting cells, up to 2% of oxygen uptake is converted into ROS (Bridges et al., 2012). Some of these intracellular ROS activate cellular signals involved in the protection against oxidative stress, such as the synthesis of glutathione (GSH), thioredoxin (Trx-1) and antioxidant enzymes. In an organism, tissues and organs contain specific antioxidant reservoirs, likely due to their different metabolic rates, which are reflected by their blood supply and oxygen consumption (Limón-Pacheco and Gonsebatt, 2009). These distinct features cause some organs, such as the brain, to be susceptible to oxidative injury.

GSH not only participates in protection against oxidative stress but also performs relevant functions in various cellular processes, such as cell proliferation, apoptosis and differentiation, and the inactivation of toxic electrophilic compounds (Valdovinos-Flores and Gonsebatt, 2013). Hence, alterations in GSH homeostasis due to impaired synthesis/recycling or increased consumption are associated with disease. In fact, aging as well as degenerative and neuropsychiatric diseases, such as Parkinson’s disease, Alzheimer’s disease, autism and schizophrenia, are associated with increased ROS production, the disruption of cellular GSH pools, and downregulation of GSH-dependent enzymes (Ballatori et al., 2009; Johnson et al., 2012; Gu et al., 2015).

Because GSH does not readily penetrate the blood-brain barrier (BBB) (Anderson et al., 1989), central nervous system (CNS) GSH levels depend on *de novo* synthesis by two enzymes: γ-glutamate-cysteine ligase (GCL) and glutathione synthetase (GS) (Valdovinos-Flores and Gonsebatt, 2012).

Glutamate-cysteine ligase catalyzes the first rate-limiting enzymatic step, which is limited by the availability of the sulfhydryl amino acid (AA) L-cysteine (L-cys) (Valdovinos-Flores and Gonsebatt, 2012). L-cystine, the disulfide form of L-cysteine, is transported across the CSF–brain barrier via the Na^+^- independent L-cystine/L-glutamate exchanger system $x_{c}^{-}$ (Shih et al., 2006). L-cystine is reduced in the BBB, and the resulting cysteine is transported out of the endothelial cells into the brain via L-type amino acid transporter 1 (LAT1) (Sánchez del Pino et al., 1995). Extracellular L-cysteine is oxidized to L-cystine and transported through the $x_{c}^{-}$ system into astrocytes (Shih et al., 2006), which release GSH via the multidrug resistance protein 1 (MRP1) as the first step in supplying cysteine for GSH synthesis in neurons (Minich et al., 2006). Neurons take up cysteine via excitatory amino acid carrier 1 (EAAC1/EAAT3), which is a glutamate/cysteine transporter that is part of the $x_{A\,G}^{-}$ system (Zerangue and Kavanaugh, 1996).

The systemic inhibition of GSH synthesis by L-buthionine-S-R-sulfoximine (BSO, Meister, 1995) in liver and kidneys or the exposure to a toxic metalloid such as arsenite, rose brain GSH pools in mice. Simultaneously, we observed an up-regulation of amino acid transporters, such as xCT and EAAT3 (Limón-Pacheco et al., 2007; Ramos-Chávez et al., 2015; Garza-Lombó et al., 2018), which were associated with a disruption of glutamate disposition and potentially leading to neurotoxicity (Nelson-Mora et al., 2018).

In the cerebellum, EAAT3 up-regulation and increased GSH levels were associated with an earlier activation of the NGF/TrkA and mTOR signaling pathways in neurons (Garza-Lombó et al., 2018).

Nerve growth factor is a neurotrophin (NT) that regulates neuronal development, differentiation, plasticity, cell death and survival (Lu et al., 2005). NGF binds to two distinct classes of transmembrane receptors, the low-affinity p75 neurotrophin receptor (p75NTR), a member of the tumor necrosis receptor superfamily, and high-affinity tropomyosin-related kinase receptor A (TrkA) (Lu et al., 2005).

In the human and murine CNS, NGF- and TrkA-positive regions correlate with the anatomical distribution of cholinergic neurons in the basal forebrain and striatum (Bizon et al., 1999). Moreover, NTs and their receptors are expressed in non-neural tissues (Yamamoto et al., 1996). The NGF/TrkA pathway participates in the induction of antioxidant responses in both the CNS and non-neural tissues (Pan and Perez-Polo, 1993, 1996; Sampath et al., 1994; Guégan et al., 1999; Salinas et al., 2003; Podratz and Windebank, 2005). Furthermore, exogenous NGF increases the activity of the major antioxidant enzymes in brain tissues and attenuates neuronal injuries induced by traumatic brain injury, such as the quinolinic acid induced decline in glutathione reductase activity and GSH content in striatum (Cruz-Aguado et al., 2000; Zhou et al., 2003).

Also, NGF synthesis and the NGF/TrkA pathway are activated by oxidative stress in non-neural tissues (Caporali et al., 2008; Wang et al., 2013). We have also gathered evidence suggesting that NGF plays a relevant role in maintaining reduced thiol levels and protecting the liver from oxidative stress and xenobiotic injury through the NGF/TrkA/PI3K/AKT/nuclear factor kappa B (NFκB) pathway (Valdovinos-Flores and Gonsebatt, 2013). In fact, transgenic mice overexpressing NGF display elevated GSH concentrations in the plasma, brain, and liver (Arsenijevic et al., 2007).

Here, we document that a systemic reduction of GSH levels, up-regulates the transcription of L-cys/L-cys_2_ and glutamate AA transporters and antioxidant genes in the striatum trough the TrkA/Akt pathway, which was mediated, at least in part, by peripheral NGF levels.

## Materials and Methods

### Chemicals and Antibodies

All chemicals were purchased from Sigma (St. Louis, MO, United States) unless otherwise indicated. The neutralizing anti-NGF antibody (Cat No. ab16161) and the TrkA (Cat. No. ab76291), proNGF (Cat. No. ab52918), transferrin (Cat No. ab82411), and NeuN (Cat. No. ab5541) antibodies were purchased from Abcam (Cambridge, MA, United States). The primary antibody against native glyceraldehyde-3-phosphate dehydrogenase (GAPDH; Cat. No. MAB374) was purchased from Millipore (Bedford, MA, United States); β-tubulin (Cat. No. T4026) from Sigma-Aldrich. Antibodies against phospho-TrkA (Tyr 490; Cat. No. 9141), Akt (Cat No. 9272), NGF (Cat. No. 2046) and nucleophosmin (NPM; Cat. No. 3542) and the secondary anti-rabbit antibody used for western blotting were obtained from Cell Signaling Technology (Danvers, MA, United States). DyLight 649-labeled Lycopersicon esculentum (Tomato) lectin (Vector Laboratories, Burlingame, CA; Cat. No DL-1178) and the Alexa Fluor 546-conjugated anti-rabbit (Invitrogen, Carlsbad, CA, United States; Cat. No. A10040) and Alexa Fluor 488-conjugated anti-mouse (Invitrogen; Cat. No. A21202) secondary antibodies were used for immunofluorescence.

### Animal Use and Treatments

Six-week-old male BALB/c mice were obtained from the animal care facility at the Instituto de Investigaciones Biomédicas at Universidad Nacional Autónoma de México. The animals were housed on a 12-h light/dark cycle. Intraperitoneal (i.p.) injections of 6 mmol kg^−1^ BSO or 14 mg kg^−1^ iAs dissolved in a 0.9% saline solution or 0.9% saline solution alone were administered to mice. Control and treated mice were sacrificed via cervical dislocation at 0.5, 2, 6, or 24 h after treatment. To examine the role of systemic NGF, the animals were pretreated with 1 mg kg^−1^ neutralizing anti-NGF antibody dissolved in saline solution 1 h prior to BSO administration and sacrificed after 2 h (Valdovinos-Flores and Gonsebatt, 2013). The striatum and liver were harvested and washed with ice-cold PBS to remove blood and tissue debris. Then, the tissues were immediately frozen in liquid nitrogen and stored at −70°C until use in RT-PCR experiments or processed immediately for protein studies.

### Glutathione Determination

Glutathione and GSH disulfide (GSSG) contents were assayed using the fluorometric o-phthalaldehyde (OPA) method (Senft et al., 2000), which was previously proven in our laboratory by HPLC [18], using 96-well black microplates. Fluorescence readings were taken at 365-nm excitation and 430-nm emission with a Beckman Coulter DTX 800/880 Multimode Detector (Beckman Coulter, Fullerton, CA, United States). The final values were calculated as the fluorescence of unit B minus the fluorescence of unit A (UF_*B*_-UF_*A*_ = UF_*F*_).

### Western Blotting

To evaluate NGF levels in the striatum and for phosphorylation levels, fresh tissue was homogenized at a 30% ratio (w v^−1^) in kinase extraction buffer A (10 mM HEPES, pH 7.9, 10 mM KCl, 10 mM EDTA, 1 mM DTT, 0.4% v v^−1^ IGEPAL, 1 mM Na_3_VO_4_, 1 mM PMSF, and 10 mg mL^−1^ aprotinin and leupeptin) using a previously described method (Kaneko et al., 2004). The samples were incubated on ice for 10 min and then centrifuged at 4000 *g* for 15 min at 4°C. The supernatant fractions (cytoplasmic soluble proteins) were collected. The nuclear pellet was washed and then lysed in buffer C (20 mM HEPES, pH 7.9, 200 mM NaCl, 1 mM EDTA, 5% v v^−1^ glycerol, 1 mM DTT, 1 mM Na_3_VO_4_, 1 mM PMSF, and 10 mg mL^−1^ aprotinin and leupeptin). The lysates were incubated on ice for 2 h and then centrifuged at maximum speed for 15 min. Protein concentrations were determined using the micro Bradford method (Bio-Rad, Hercules, CA, United States), according to the manufacturer’s protocol. Peripheral blood was collected after the mice were sacrificed via cervical dislocation; the blood was stored at 4°C overnight and then centrifuged at 4000 *g* for 15 min at 4°C to obtain plasma. The supernatant fractions were collected. Protease inhibitors (1 mM PMSF and 10 mg mL^−1^ aprotinin and leupeptin) were added to the plasma fraction and samples were stored at −70°C until the Western blot analysis. Lysates and plasma samples were separated on 10% SDS–PAGE gels and transferred to a Hybond-ECL nitrocellulose membrane (Amersham Biosciences, Piscataway, NJ, United States). Membranes were blocked overnight at 4°C with Tris-buffered saline containing 5% non-fat milk and then incubated with the primary antibody against the phosphorylated kinase. An antibody against the native form was used to detect the total protein content. For the analysis of transcription factor translocation, both the nuclear and cytoplasmic extracts were utilized, and NPM and GAPDH, respectively, were used as loading controls. For the evaluation of the plasma and striatal NGF levels, ferritin and β-tubulin, respectively, were used as loading controls. Proteins were visualized via chemiluminescence using the ECL Advance Western Blotting Detection Kit (Amersham Biosciences), and images were captured and analyzed by densitometry using a Kodak ID Version 3.6 Image Analyzer (Kodak, NY, United States).

### Immunofluorescence Staining

Control and BSO-treated mice were transcardially perfused with a cold saline solution followed by buffered paraformaldehyde (4%). The brains of the mice were removed, postfixed with the same fixative for 24 h at 4°C, and then cryoprotected using a graded series of buffered sucrose solutions (20% and 30%). Sections (50 μm thick) were cut using a cryostat and individually collected in 96-well culture plates filled with 0.1 M phosphate buffer (PB). After three washes with PB supplemented with 0.3% Triton X-100 (PBT), the sections were incubated overnight at 4°C in PBT containing 0.3% BSA (Santa Cruz), 2% normal horse serum (Vector Laboratories), and the mouse anti-NeuN and rabbit anti-TrkA or anti-NGF primary antibodies (at a dilution of 1:200 in all cases except for NeuN, which was used at a dilution of 1:600). After three washes with PBT, the sections were incubated with the Alexa Fluor 546-conjugated anti-rabbit (1:600) and Alexa Fluor 488-conjugated anti-mouse (1:400) secondary antibodies for 2 h at room temperature in the dark. Then, the sections were washed with 0.1 M PB, endothelial cells were counterstained with lectin, and nuclei were counterstained with 4′,6-diamidino-2-phenylindole (DAPI, Invitrogen), according to the manufacturer’s protocols. Finally, liver sections were mounted in Fluorescent Mounting Medium (DAKO, Glostrup, Denmark). Fluorescence images were sequentially acquired with a Nikon A1R+ laser scanning confocal Eclipse Ti-E inverted microscope (Nikon Corporation, Tokyo, Japan) equipped with a motorized stage (TI-S-E, Nikon). Imaging was performed with a CFI Plan Apo VC 60xA WI DIC N2 objective (N.A. 1.2) using 3 mW of 405 nm laser power, 0.77 mW of 488 nm laser power, 0.7 mW of 561 nm laser power and 125 mW of 647 nm laser power, a pinhole aperture of 12.77 mm and both PMT and GaAsP detectors, all of which were controlled using NIS Elements C software v4.50 (Nikon). ImageJ version 1.46r software (U.S. National Institutes of Health, Bethesda, MD, United States) was used to analyze the final images.

### Real-Time RT-PCR Analysis

Total RNA was isolated using TRIzol (Invitrogen). RNA concentrations were determined by measuring the absorbance at 260 nm. RNA integrity was verified via electrophoresis on 1% agarose gels. One microgram of total RNA was reverse-transcribed to cDNA using M-MLV reverse transcriptase (Invitrogen). For the real-time RT-PCR analysis, a Rotor-Gene Q PCR cycler (Qiagen GmbH, Hilden, Germany) was used. Previously prepared cDNA was diluted 1:125 and used as a template for real-time PCR. PCR products were detected using the Kapa SYBR FAST qPCR kit (Kapa Biosystems, Woburn, MA, United States). Each reaction included 8 μL of diluted cDNA, 10 μL of Kapa SYBR FAST qPCR kit reagents and 1 μl each of 5 μM stocks of the forward and reverse primers in a total reaction volume of 20 μL. The following reaction conditions were used: an initial cycle at 94°C for 3 min, 30 cycles of 94°C for 5 s and 60°C for 20 s and a final melting curve from 73°C to 93°C to ensure that only one product was amplified. The primers used are shown in Table 1. The mean amplification efficiency ± standard deviation (SD) was 0.995 ± 0.011, and the mean correlation coefficient ± SD was 0.992 ± 0.006. The results were analyzed using the 2^–ΔΔ^CT method with *gapdh* as a reference gene (Livak and Schmittgen, 2001).

**TABLE 1:** Primers used for PCR and real-time PCR.

**Gene**	**Primers**	**Product (bp)**	**Acc. No.**	**References**
*lat1 (Slc7a5)*	F: 5′ AGC TTC TTC AAC TGG CTG TGT 3′ R: 5′ AGA GGC AGG CCA GGA TAA A 3′	133	NM_011404.3	
*xct (Slc7a11)*	F: 5′ AGC CAG TCG GTG ATA GCA AAG 3′ R: 5′ AGG GGG AAA AAC AAA ACA AGA C 3′	122	NM_011990.2	Limón-Pacheco et al.,2007
*eaac1 (Slc1a1)*	F: 5′ CGC AAA CGT CAG TGC TCA 3′ R: 5′ CCA CGA CTC CTA AGA CAA TTC C 3′	132	NM_009199.2	
*gapdh (gapdh)*	F: 5′ ACC ACC AAC TGC TTA GCC CC 3′ R: 5′ CAG CTC TGG GAT GAC CTT GC 3′	216	NM_008084.2	
*gclm (gclm)*	F: 5′ CAG TTG GAG CAG CTG TAT CAG T 3′ R: 5′ CAG TCA AAT CTG GTG GCA TC 3′	91	NM_008129.4	
*nrf2 (Nfe2l2)*	F: 5′ CAC CAG TGG ATC CGC CAG CTA 3′ R: 5′ TAT CCA GGG CAA GCG ACT CA 3′	134	NM_010902.3	

### Data Analysis

Each assay was performed in triplicate unless otherwise indicated. Data are presented as the means ± S.E., and significance was analyzed using one-way ANOVA with Tukey’s *post hoc* analysis, as indicated in each case. A *p*-value ≤ 0.05 was considered significant in all cases.

### Ethics Statement

The experiments reported in this manuscript were conducted according to the guidelines stated in the Principles of Laboratory Animal Care (Institute for Laboratory Animal Research., 2011) and the Norma Oficial Mexicana de la Secretaría de Agricultura, Ganadería, Desarrollo Rural, Pesca y Alimentación (SAGARPA, México) Especificaciones técnicas para la producción, Cuidado y uso de los animales de laboratorio (Agricultura y Ganadería, 2001).

## Results

### NGF Protein Levels Are Increased in the Plasma and Striatum, and NGF/TrkA Pathway Is Activated in the Striatum After BSO Treatment Which Depletes Liver GSH

**FIGURE 1:**
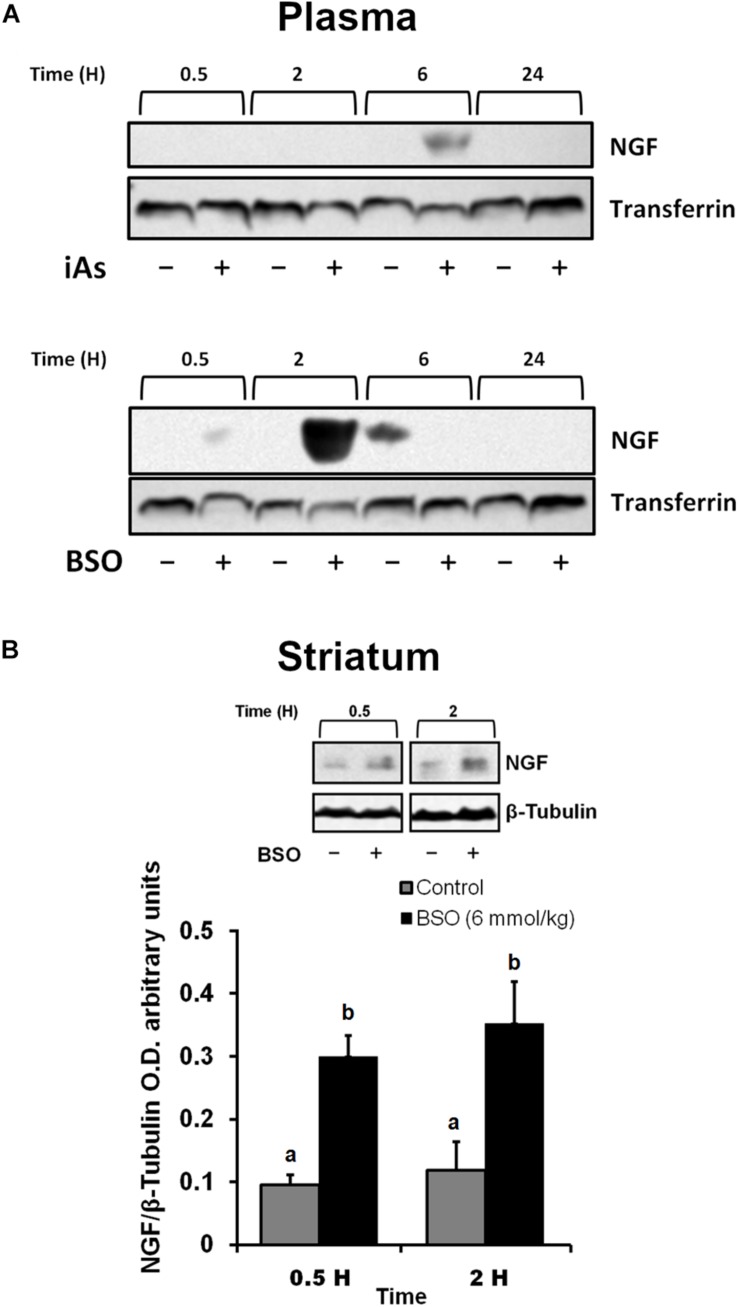
L-Buthionine-S-R-sulfoximine or iAs i.p. injection increased NGF protein levels in the plasma, and BSO treatment increased NGF protein levels in the striatum. **(A)** Increased NGF protein levels in the plasma. Western blots were performed using transferrin as a loading control. The upper image is a representative western blot of NGF in the plasma of mice treated with 14 mg kg^–1^ iAs or saline solution. The lower image is a representative western blot of NGF in the plasma of mice treated with 6 mmol kg^–1^ BSO or saline solution. **(B)** Increased NGF protein levels in the striatum. The upper panel shows a representative western blot of NGF in the striatum. Densitometric evaluations of the western blot images were performed using β-tubulin as a loading control. The bars represent the mean ± S.E. of triplicate experiments and were calculated from the densitometry data (*n* = 9). The data were analyzed using one-way ANOVA with Tukey’s *post hoc* analysis. Different letters in the superscript above each column indicate statistically significant differences; *P* ≤ 0.05.

The protein expression of *ngfb* was investigated in the plasma after the intraperitoneal (i.p.) administration of BSO or iAs. Both agents diminish GSH levels (Rodríguez et al., 2005; Valdovinos-Flores and Gonsebatt, 2013 and Table 2). Increased levels of NGF were observed in the plasma from 0.5 to 2 h after BSO injection and 6 h after iAs administration (Figure 1A), suggesting the participation of tissues in a systemic response. In fact, under the same experimental conditions, *ngfb* transcription and the TrkA/PI3K/Akt/NFκB pathway are activated in the liver (Valdovinos-Flores and Gonsebatt, 2013).

**TABLE 2:** L-Buthionine-S-R-sulfoximine modulates the redox state in the liver.

**Treatment**	**GSH (μmol/g tissue)**	**GSSG (μmol/g tissue)**	**GSH/GSSG**
Control	7.296 (±0.312)	0.134 (±0.0101)	54.193 (±3.524)
BSO (6 mmol/kg)	3.521 (±0.312)^*^	0.127 (±0.0090)	27.653 (±0.979)^*^

GSH and GSSG levels in the liver after 2 h of 6 mmol/kg of and i.p. BSO injection. Control mice were injected with a saline solution.Means (±S.E.) *n* = 5. Student’s *t*-test. The symbol “^*^” indicates statistical significance difference vs. control; *P* ≤ 0.05.

**FIGURE 2:**
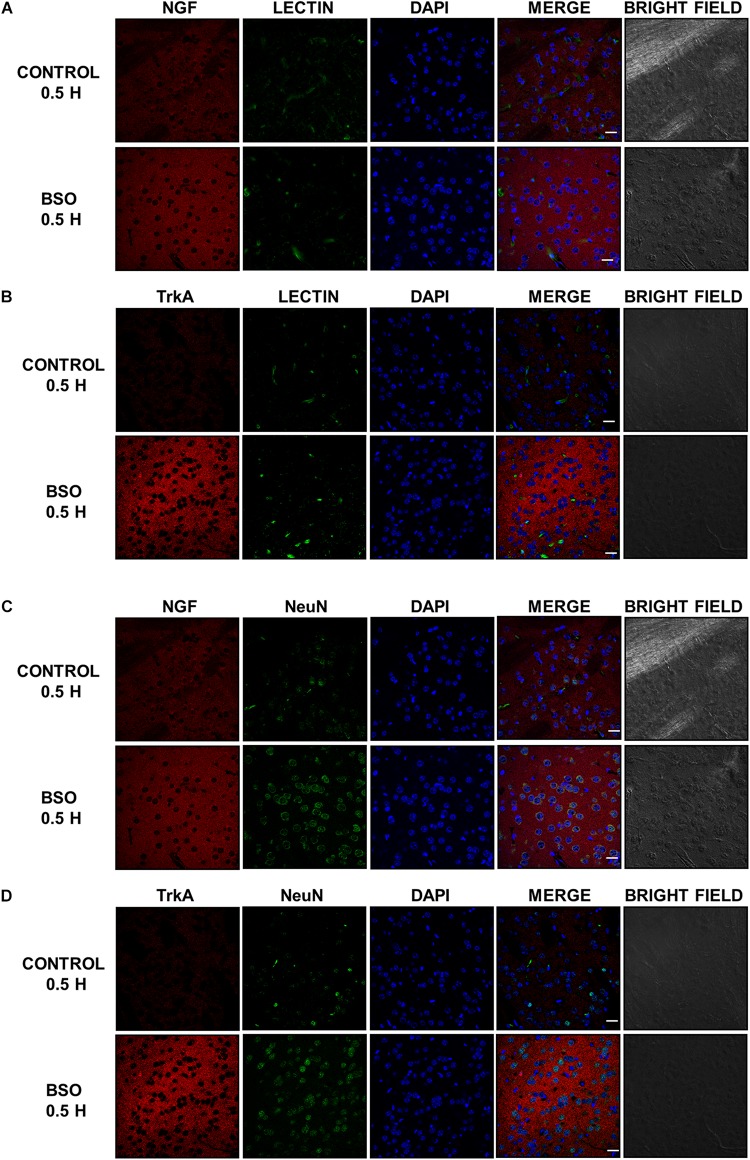
The i.p. injections of BSO increased the levels of the NGF and TrkA proteins in the mouse striatum. Immunostained and bright field confocal images of striata sections of control and BSO-injected mice. **(A)** Images of immunofluorescence staining of NGF (red) endothelial cells (lectin, green), and nuclei (DAPI, blue) in control and treated mice. **(B)** Images of immunofluorescence staining of endothelial cells (lectin, green), TrkA (red), and nuclei (DAPI, blue) in control and treated mice. **(C)** Images of immunofluorescence staining of NGF (red), neurons (NeuN, green), and nuclei (DAPI, blue) in control and treated mice. **(D)** Images of immunofluorescence staining of TrKA (red), neurons (NeuN, green), and nuclei (DAPI, blue) in control and treated mice. Scale bar: 20 microns.

**FIGURE 3:**
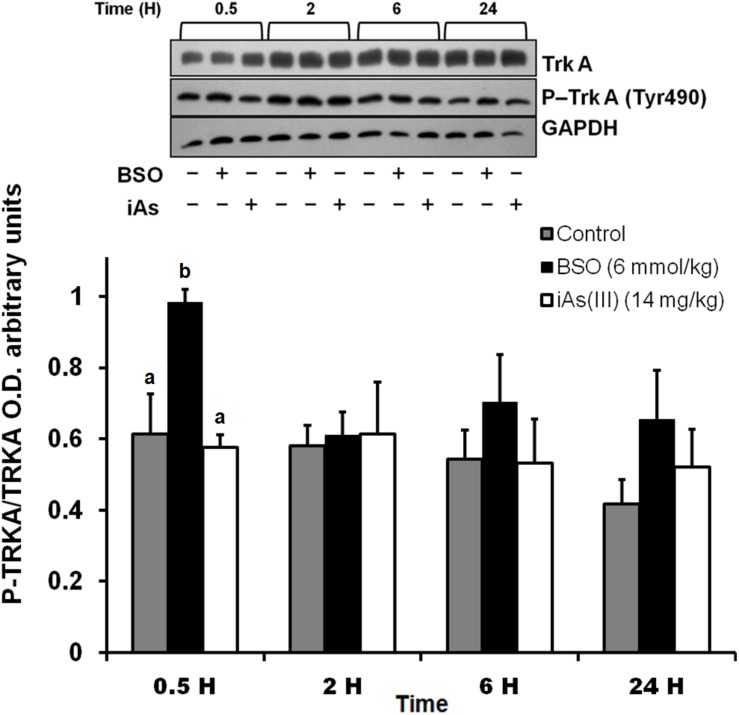
The i.p. injections of BSO activated the NGF/TrkA pathway in the striatum. Activation of the TrkA protein in the striatum. The upper panel shows a representative western blot for TrkA phosphorylated at Tyr 490 in the striatum. Densitometric evaluations of the western blot images were performed using total TrkA as a loading control. The bars represent the means ± S.E. of triplicate experiments, which were calculated from the densitometry data (*n* = 9). The data were analyzed using one-way ANOVA with Tukey’s *post hoc* analysis. Different letters in the superscript above each column indicate statistically significant differences; *P* ≤ 0.05.

In addition, we observed an upregulation of NGF protein in the striatum at 0.5 and 2 h after BSO injection. Increased NGF and TrkA protein levels in striatal neurons were confirmed at 0.5 h via immunofluorescence (Figure 2). Furthermore, increased TrkA phosphorylation was detected from 0.5 h after BSO injection (Figure 3), suggesting that the TrkA pathway is activated, despite the impermeability of BSO to the BBB. The response observed here was according to the previous report. Under the same exposure protocol, we observed the activation of the NGF/TrkA pathways in the cerebellum (Garza-Lombó et al., 2018).

These results indicate that systemic treatment with BSO induces the synthesis of NGF by peripheral tissues, as well as the NGF and TrkA protein levels and TrkA phosphorylation in the striatum, possibly as a response to systemic oxidative stress induced by GSH depletion after BSO treatment (Table 2), suggesting that NGF is a redox sensor in both the peripheral tissues and striatum.

### Peripheral NGF “Informs” the CNS (Striatum) of Systemic Oxidative Stress

The results described above suggest that NGF is positively modulated systemically in peripheral tissues and in the striatum after an oxidative insult. Thus, changes in peripheral NGF levels may constitute a molecular signal that induces the NGF/ TrkA pathway in the striatum (CNS). Consequently, we investigated whether peripheral NGF was responsible for these effects on the striatum.

**FIGURE 4:**
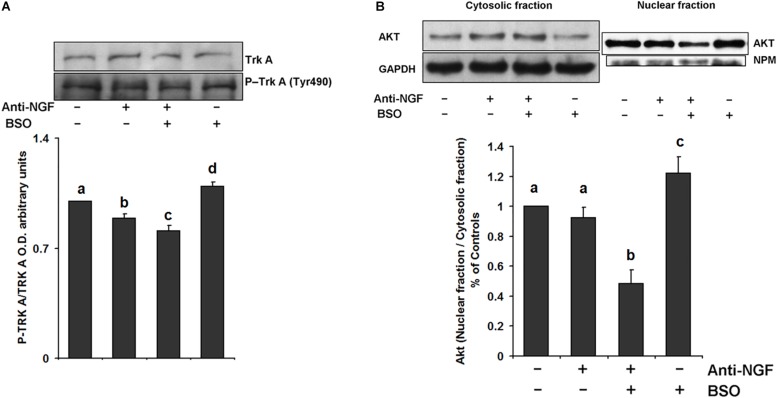
Pretreatment with a neutralizing anti-NGF antibody abrogated the response of the TrkA/PI3K/Akt/ pathway. For the western blot analysis, the mice were pretreated for 1 h with 1 mg kg^–1^ neutralizing anti-NGF antibody and sacrificed 2 h after the i.p. injection of 6 mmol kg^–1^ BSO. Total proteins were extracted from the striata of treated mice, as described in the Methods section. The upper panels show representative western blots of **(A)** TrkA phosphorylated at Tyr 490 and **(B)** Akt nuclear translocation in the striatum. Densitometric evaluation of the western blot images was performed using the following loading controls: **(A)** total TrkA and **(B)** GAPDH for the cytosolic fraction and NPM for the nuclear fraction. The bars represent the means ± S.E. of triplicate experiments, which were calculated from the densitometry data (*n* = 9). The data were analyzed using two-way ANOVA with Tukey’s *post hoc* analysis. Different superscript letters above each column indicate statistically significant differences; *P* ≤ 0.05.

We evaluated the effect of neutralizing peripheral NGF using an anti-NGF antibody that does not permeate the BBB as a pretreatment before the i.p. injection of BSO. Mice were treated with a neutralizing anti-NGF antibody for 1 h prior to the BSO injection and sacrificed 2 h later. In these animals, TrkA phosphorylation, Akt nuclear translocation were prevented in the striatum (Figure 4). Suggesting that peripheral NGF is a critical modulator of the NGF/TrkA/Akt/ pathway in the striatum.

### The Expression of the L-cys and L-cys2/Glutamate Amino Acid Transporters mRNA Is Upregulated in the Striatum After BSO and Is Mediated by the NGF/TrkA/Akt/Nrf2 Pathway

**FIGURE 5:**
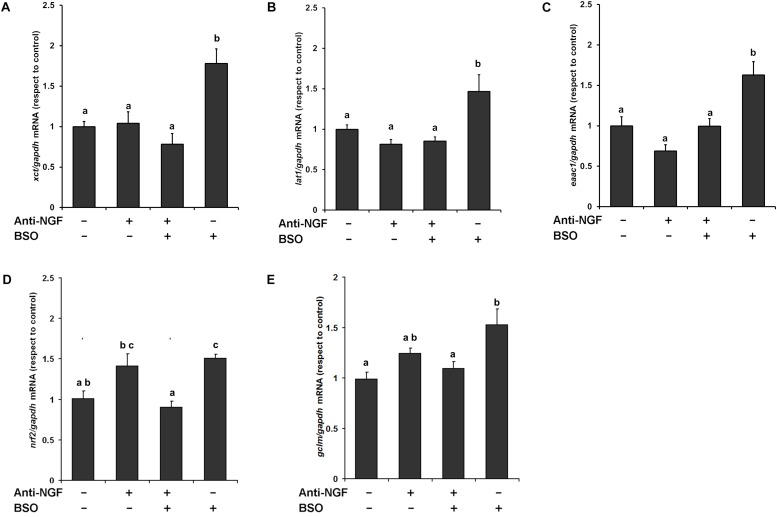
L-Buthionine-S-R-sulfoximine treatment increased the expression of the AA transporter genes, as well as genes related to antioxidant enzymes and the pretreatment with a neutralizing anti-NGF antibody, inhibited that response. For Real-time PCR, total RNA was extracted from the striatum and cDNAs were synthesized using procedures described in the Methods section. Levels of the **(A)** *xct*, **(B)** *lat1*, **(C)** *eaac1*, **(D)** *nrf2*, and **(E)** *gclm* mRNAs were quantified in the striata of mice that had been pretreated for 1 h with 1 mg kg^–1^ neutralizing anti-NGF antibody and sacrificed 4 h after the i.p. injection of 6 mmol kg^–1^ BSO. The data were normalized to the *gapdh* mRNA. The vertical bars indicate the mRNA levels relative to the control group. The bars represent the means ± S.E. of triplicate experiments (*n* = 9). The data were analyzed using two-way ANOVA with Tukey’s *post hoc* analysis. Different superscript letters above each column indicate statistically significant differences; *P* ≤ 0.05.

We previously found that *xct* mRNA is upregulated in whole brain homogenates after GSH depletion (Limón-Pacheco et al., 2007), and more recently we have shown that EAAT3 protein levels are upregulated after the i.p. injection of BSO via the NGF/TrkA and mTOR signaling in the cerebellum (Garza-Lombó et al., 2018). Thus, we next examined the modulatory effects of BSO on the mRNA expression of CNS AA transporters that provide L-cys, such as *lat1* and *eaac1*, and L-cys_2_/glutamate, such as *xct*, in the striatum. A significant increase in mRNA levels was detected for all the genes evaluated after BSO injection (Figure 5). These results show that BSO treatment modulates the levels of the transcripts encoding AA transporters related to L-cys/L-cys_2_ availability in the striatum.

Furthermore, the anti-NGF antibody pretreatment abrogated the BSO-induced changes in the levels of the *xct*, *lat1*, and *eaac1* mRNA (Figure 5). In addition, the anti-NGF antibody pretreatment abolished the previously observed changes in the levels of the *gclm* and *nrf2* mRNA’s (Limón-Pacheco et al., 2007). Thus, peripheral NGF is a critical modulator of the NGF/TrkA/Akt/Nrf2 pathway in the striatum and participates in modulating the transcription of *gclm*, *nrf2*, and genes involved in AA transport systems that are responsible for the uptake of L-cys/L-cys_2_ in the striatum in different cell types such as endothelial, astrocytes, microglia, and neurons.

## Discussion

Glutathione is an essential peptide not only for the cellular redox homeostasis but also for the metabolization/elimination of metabolites and chemicals including drugs. Environmental exposure to metals, pesticides, ionizing radiation, U.V., or some pharmaceutical drugs, diminishes GSH pools in target tissues (Limón-Pacheco and Gonsebatt, 2009). In the CNS, low levels of GSH have been associated with neuropsychiatric disorders such as Parkinson’s and Alzheimer’s disease, and schizophrenia (Ballatori et al., 2009; Do et al., 2009). Here, we used a murine model to explore the protective response observed in the CNS after a systemic inhibition of GSH synthesis.

The role of NGF in the CNS has been studied and reviewed extensively. Furthermore, NGF and its receptors are expressed, and in many cases, modulated during stress in non-neural tissues, such as epithelial cells, fibroblasts, lymphocytes, activated macrophages, the pancreas, the heart and the liver (Yamamoto et al., 1996; Micera et al., 2001; Caporali et al., 2008; Abram et al., 2009). Moreover, it has been demonstrated that NGF levels are increased in the plasma after social and physical stress (see a previous review: Alleva and Santucci, 2001). These studies suggest a systemic role for this NT as a neuro-immune-endocrine modulator with fundamental functions in the regulation of homeostatic processes, as was first proposed by Levi-Montalcini and Aloe (1997).

Previous *in vivo* and *in vitro* studies have revealed a role for NGF in the activation of the antioxidant response in the CNS in regions such as the cerebellum (Pan and Perez-Polo, 1993; Guégan et al., 1999; Garza-Lombó et al., 2018). This cell survival response occurs as a consequence of the activation of the TrkA receptor (Sofroniew et al., 2001; Lu et al., 2005). The pathways activated by TrkA include Ras, Rac, PI3K, and PLC-γ1, which in turn activate mitogen-activated protein kinase (MAPK) cascades and Akt to generate inositol triphosphate and diacylglycerol (Sofroniew et al., 2001).

On the other hand, the p75^*NTR*^ receptor has been reported to either induce or prevent apoptosis upon oxidative insult in both *in vivo* and *in vitro* models. Nevertheless, Tyurina et al. (2005) showed that the role of the p75^*NTR*^ antioxidant response is not related to its extracellular receptor domain or to extracellular ligands of this NT receptor. Moreover, the increase in GSH levels induced by Nrf2 activation prevents the p75^*NTR*^-mediated apoptosis of neurons *in vitro* (Vargas et al., 2006).

In our mouse model, BSO and iAs up-regulated NGF protein levels in plasma although in the case of iAs, at a delayed time scale (Figure 1) that was not associated with TrkA activation in the striatum (Figure 3). These results suggest that NGF synthesis is modulated by changes in the redox cellular state, despite the differences in the time scales of these responses, probably due to the specific characteristics of the target organ, drug mechanisms of action and kinetic disparities. Because we were searching for early responses, we decided to continue only with BSO treatments.

Similarly to the results obtained here, earlier works have reported oxidative stress-related NGF up-regulation in peripheral tissues such as heart (Caporali et al., 2008) and liver (Valdovinos-Flores and Gonsebatt, 2013), that could lead to the observed increase in plasma NGF protein levels after treatment with BSO and iAs (Figure 1).

L-Buthionine-S-R-sulfoximine also induced the expression of NGF and TrkA protein levels in the striatum, as well as TrkA phosphorylation at Tyr 490 (Figures 1, 2, 3). These results suggest that NGF levels are positively modulated systemically in peripheral tissues and the striatum after an oxidative insult. Thus, changes in peripheral NGF levels may constitute a molecular signal to induce NGF and TrkA transcription in the striatum (CNS). To evaluate this hypothesis, we inhibited the NGF/TrkA pathway by using an anti-NGF antibody, which does not permeate the BBB, as a pretreatment before the i.p. injection of BSO. The administration of a neutralizing anti-NGF antibody abrogated signaling through the NGF/TrkA/Akt pathway in the liver in our previous study (Valdovinos-Flores and Gonsebatt, 2013). Here, the neutralizing anti-NGF antibody prevented the activation of the NGF/TrkA/Akt pathway in the striatum (Figure 4), according to our hypothesis.

The fact that transgenic mice overexpressing NGF exhibit elevated GSH concentrations in the brain, plasma, and liver (Arsenijevic et al., 2007) supports our hypothesis that NGF plays a key role as a systemic redox sensor in both the CNS and peripheral tissues and integrates homeostatic systemic responses. In that respect, we have observed that agents that diminish the levels of cellular GSH, such as BSO, iAs, and APAP, induce *ngfb* transcription in the mouse liver. Additionally, the use of a neutralizing anti-NGF antibody diminished GSH levels and downregulated *trx-1* mRNA levels in the liver (Valdovinos-Flores and Gonsebatt, 2013).

Glutathione does not penetrate the BBB easily (Anderson et al., 1989), therefore GSH synthesis in the brain is limited by the availability of the sulfhydryl AAs L-cys and L-cys_2_ (Shih et al., 2006). We hypothesized that the activation of the NGF/TrkA signaling pathway in the striatum after BSO, might be associated with the transcriptional upregulation of the amino acid transporters genes related to the availability of L-cys and L-cys_2_.

Thus, we evaluated the modulation of the *xct*, *lat1* and *eaac1* mRNA levels in the striatum. BSO treatment increased the transcription of these transporters (Figure 5). *In vivo* and *in vitro* models, the modulation of xCT and EAAC1 expression by oxidative stress stimuli has been widely reported (Kim et al., 2001; Muguruma et al., 2007; Escartin et al., 2011; Ramos-Chávez et al., 2015; Nelson-Mora et al., 2018). The genes that encode xCT, EAAC1 and LAT1 contain putative antioxidant response element (ARE) motifs in their proximal promoter sequences (Diah et al., 2001; Sasaki et al., 2002; Escartin et al., 2011), suggesting that the transcription factor Nrf2 participates in the upregulation of the transcription of the AA transporters. In fact, we found an upregulation of *nrf2* mRNA levels in response to BSO treatment (Figure 5) as was previously reported under the same experimental conditions (Limón-Pacheco et al., 2007). It has been reported that *nrf2* mRNA levels correlated with its protein levels and have been observed as part of antioxidant enzyme regulation (Frohlich et al., 2008). Another antioxidant gene positively modulated include the gene that codified to the regulatory subunit of the first rate-limiting enzyme of glutathione synthesis, *gclm*, which mRNA level was increased after BSO treatment.

This finding is consistent with the observations reported by Kosaka et al. (2010) and Mimura et al. (2011), who showed that TrkA activation via NGF induces the nuclear translocation of Nrf2 and subsequently induces the transcription of ARE-containing genes, including *ngfb*, in PC12 cells. Notably, the mRNA increase of all the genes evaluated was abolished by the use of a neutralizing anti-NGF antibody (Figure 5).

The induction of the L-cys_2_/L-glu exchange transporter, which is mediated by system $x_{c}^{-}$, represents potentially a source of excitotoxic extracellular L-glu in the striatal parenchyma (Bridges et al., 2012; Ramos-Chávez et al., 2015; Nelson-Mora et al., 2018). Thus, we evaluated whether, along with *xct*, the levels of the *glt-1* mRNA, which is a member of the system $x_{A\,G}^{-}$ transporter and is important for the clearance of L-glu (Pines et al., 1992), were increased. However, we did not detect any changes in the transcription of *glt-1* after treatment with BSO (data not shown), suggesting that *xct* upregulation could lead to increased extracellular glutamate levels, as observed in mice that have been gestationally exposed to arsenic (Ramos-Chávez et al., 2015; Nelson-Mora et al., 2018).

Because NGF induces an antioxidant response via TrkA/Akt or MAPK signaling pathway, these data support our previous finding that increased levels of GSH in the brain are associated with ERK2 activation (Limón-Pacheco et al., 2007), as well as the association of TrkA and mTOR signaling pathways with the increased levels of the EAAC1 protein in the cerebellum (Garza-Lombó et al., 2018).

Our findings suggest that systemic NGF helps to maintain redox homeostasis, which would explain why NGF and TrkA levels are increased in both the plasma and brain during the systemic oxidative stress generated by physical activity (Alleva and Santucci, 2001; Ang et al., 2003; Chung et al., 2010), as well as the neuroprotective effect of physical activity on murine models of brain damage (Ding et al., 2004; Matsuda et al., 2011). However, an increase in plasma NGF levels due to excess physical activity is also associated with the occurrence of a Th2 response in subjects with allergic diseases (Bonini et al., 2013). Moreover, the experimental use of NGF antibodies as pain-relieving drugs might cause joint destruction and autonomic dysfunction by inducing changes in GSH homeostasis (Miller et al., 2017; Mullard, 2018).

**FIGURE 6:**
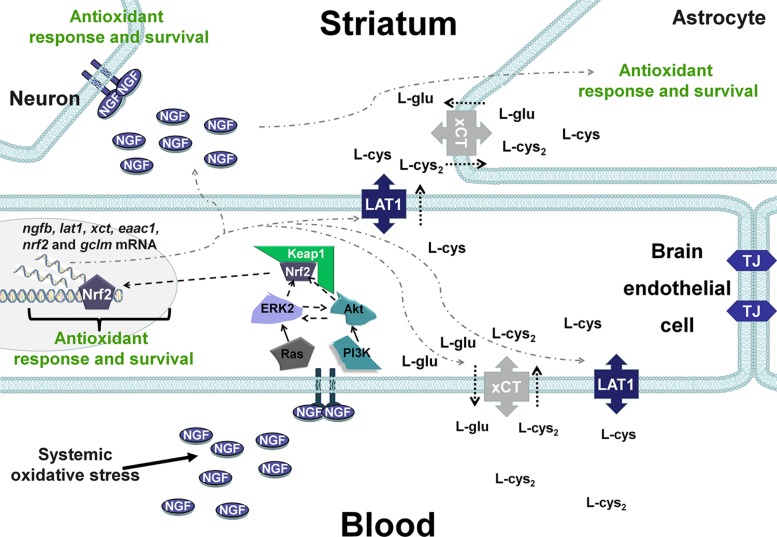
Hypothetical scenarios for the role of NGF after systemic oxidative stress. NGF levels increase in peripheral tissues and is secreted into the circulation, resulting in autocrine/paracrine effects. Peripheral NGF activates the TrkA pathway in brain endothelial cells, which induces the transcription of transporters of the sulfhydryl AAs L-cys/L-cys2 in neurons and glial cells. In addition, NGF synthesis is increased in brain endothelial cells (BECs), and NGF is secreted into the brain parenchyma, activating the NGF/TrkA/PI3K/Akt/Nrf2 pathway that increases the expression of antioxidant genes in the CNS. TJ, tight junctions.

Taking into account these results and our previous studies (Limón-Pacheco et al., 2007; Valdovinos-Flores and Gonsebatt, 2013; Garza-Lombó et al., 2018), it is possible that changes in the systemic redox state induce tissue-specific responses through Nrf2 or NF-κB that include the synthesis of NGF, which increases circulating NGF levels in the blood. Then, although at the time evaluated here it was not observed, it is possible that peripheral NGF activates the NGF/TrkA signaling pathway in brain endothelial cells and enables the entrance of the sulfhydryl AAs L-cys/L-cys2 into the brain parenchyma and the subsequent secretion of NGF in the brain, where it plays a critical role in the antioxidant response via the NGF/TrkA pathway (Figure 6) in neurons and glial cells. In fact, xCT mRNA and protein expression have been shown along brain blood vessels, including endothelial cells, meninges and astrocytes, EAAC1 is expressed in neurons and LAT1 in microvascular cells of the BBB and neurons (Burdo et al., 2006; Muller and Heuer, 2014; Ottestad-Hansen et al., 2018). Another possibility is that peripheral NGF could enter directly to the CNS as was proposed for (Levi-Montalcini and Aloe, 1985), although the mechanisms underlying this phenomenon are unknown.

The present study supports a systemic role for this NT as a neuro-immune-endocrine modulator, with fundamental functions in the regulation of homeostatic processes, as proposed by Levi-Montalcini (Levi-Montalcini and Aloe, 1997). However, further investigations of the specific responses of other organs to NGF are needed, because the increased expression of NGF is associated with allergic and inflammatory reactions in the lungs and the bladder, respectively (Pines et al., 1992; Guerios et al., 2006; Bonini et al., 2013). In addition, the upregulation of *xct* expression in astrocytes might increase extracellular glutamate concentrations in some brain regions, generating excitotoxicity (Nelson-Mora et al., 2018).

## Conclusion

In conclusion, we provide evidence that changes in the redox cellular state induce increases in peripheral NGF levels that orchestrate a neuroprotective response in the CNS, at least in the striatum, through the NGF/TrkA/Akt/Nrf2 pathway, including increased expression of genes involved in the uptake of the AAs L-cys and L-cys_2_ and *gclm*, all of which are related to GSH synthesis and transport from the blood to the neuronal parenchyma. Recently, the hNGPp peptide that harbors a mutation reducing the pro-nociceptive activity of NGF, delivered intranasally in mice, activated the TrkA and downstream Erk1/2, Akt pathways leading to neuroprotective and anti-amyloidogenic actions (Cattaneo and Capsoni, 2019). These findings prompt further studies of the role of peripheral NGF in the modulation of GSH homeostasis in the CNS and generate new perspectives for therapies that target ROS in patients with neurodegenerative diseases.

## Data Availability

All datasets generated for this study are included in the manuscript and/or the supplementary files.

## Ethics Statement

The animal study was reviewed and approved by The CICUAL Comission at the Instituto de Investigaciones Biomédicas, UNAM.

## Author Contributions

CV-F designed the study, performed the experiments, and prepared the manuscript draft. JHL-P and CG-L contributed with important intellectual inputs. RL-R performed Western blot analysis for NGF determination in plasma. PP helped in animal treatments and protein determinations. MEG designed the study, applied for approval from the Research Ethics Board, and reviewed the manuscript draft.

## Conflict of Interest Statement

The authors declare that the research was conducted in the absence of any commercial or financial relationships that could be construed as a potential conflict of interest.
